# Supplementary material for: Prototype of a bistable polariton field-effect transistor switch
Source: Sci Rep. 2017 Jul 11;7:5114. doi: 10.1038/s41598-017-05277-1 (PMC5506067; doi:10.1038/s41598-017-05277-1)
Supplement: Supplementary file 1 — Supplementary Information [file 41598_2017_5277_MOESM1_ESM.pdf]

Supplementary Information

Prototype of a bistable polariton field-effect transistor switch

H. Suchomel[1,+], S. Brodbeck[1,+], T.C.H. Liew[2], M. Amthor[1], M. Klaas[1], S. Klembt[1], M. Kamp[1],S. Höfling[1,3], C. Schneider[1,*]

[1]*Technische Physik and Wilhelm-Conrad-Röntgen-Research Center for Complex Material Systems, Universität Würzburg, Am Hubland, 97074 Würzburg, Germany.*
[2]*Division of Physics and Applied Physics, School of Physical and Mathematical Sciences, Nanyang Technological University, 637371 Singapore, Singapore.*
[3]*SUPA, School of Physics and Astronomy, University of St. Andrews, St. Andrews, KY 16 9SS, United Kingdom.*
[+]*These authors contributed equally to this work.*
**christian.schneider@physik.uni-wuerzburg.de*

In the following, additional experimental data and details on the demonstration of a bistable polariton field-effect transistor switch will be provided.

Optical characterization

Initially, the planar, unprocessed microcavity sample has been characterized via low temperature white-light reflectance measurements. By utilizing the intrinsic layer thickness gradient along the radial direction of the wafer the cavity mode can be tuned through the exciton energy resonance. Fig. S1a depicts the energies of the absorption dips from the reflectance spectra, which we relate to the lower (LP) middle (MP) and upper (UP) polariton branches, due to the coupling with the heavy and light hole exciton modes. Both the LP and the MP show a clear anti-crossing with the respective higher polariton branch which is the fingerprint for strong coupling between the cavity mode and the QW excitons. The inset shows the reflectance spectrum around resonance between the LP and MP branch. From the spectra, we extract a Rabi-splitting of (8.7 ± 0.1) meV.

The etched microwire sample has been characterized under open circuit conditions by investigating the power dependent emission spectra in a momentum resolved spectroscopy setup. Fig. S1b depicts the emission intensity as well as the evolution of the linewidth at the relevant in-plane wave vector where condensation takes place around ~ $k_{||}$ = 1 µm^{-1} as a function of the pump power. Data have been acquired on the processed sample, approximately 50 µm away from the contact. From the non-linearity, we can extract a threshold power for polariton lasing of (4.5 ± 0.5) mW. Furthermore, Fig. S1c depicts the power dependent emission energy of the relevant in-plane wave vector where condensation takes place around ~ $k_{||}$ = 1 µm^{-1}. The continuous blue shift above the threshold is a clear indicator that strong coupling conditions persist in our processed wire up to 20 mW.

Power dependent photoluminescence was investigated via momentum resolved spectroscopy. Using a standard coupled oscillator model by coupling the three lowest lateral cavity modes with the heavy hole exciton to fit the spectra gives a heavy hole exciton-photon detuning of the ground state of δ = -13.6 meV. From this calculation one gets the Hopfield coefficients for the exciton and the three lateral cavity photon fractions in the ground state of the system. At zero in-plane wave vector the exciton fraction amounts to approximately 8%. The quite large negative exciton-photon detuning at open circuit conditions was chosen because an applied electric field leads to a renormalization of the polariton energy levels [1]. Therefore, with increasing electric field strength the exciton-photon detuning shifts towards smaller absolute values and thereby the exciton fraction in the lower polariton branch increases. Moreover, the propagating polariton condensate carries an in-plane wave vector unequal zero which leads additionally to an increased exciton fraction compared to the value at zero in-plane wave vector. According to that the expected polariton lifetime is a complex function of field strength, position on the wire and pump power leading to an estimated polariton lifetime between 3 and 10 ps.

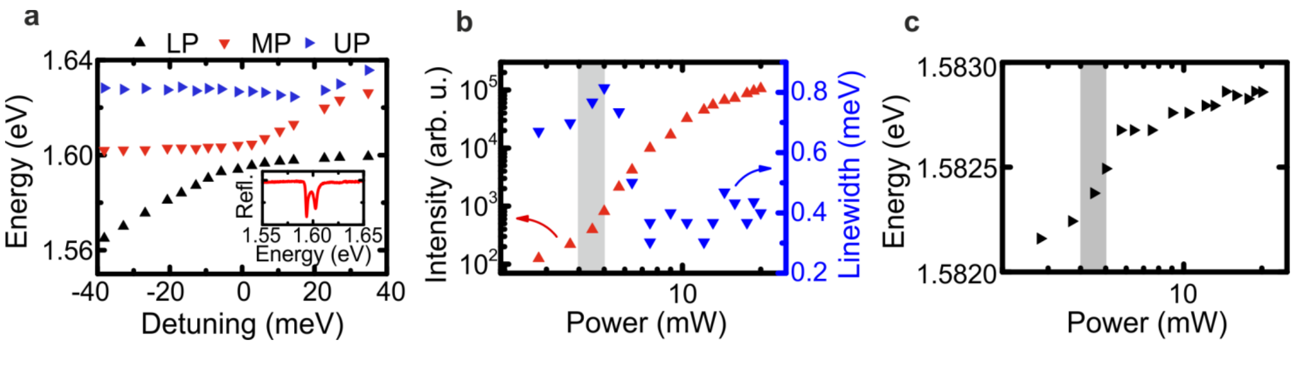

Figure S1 | Optical characterization for strong coupling and polariton condensation. (**a**) Energy position of the absorption dips as a function of exciton-photon detuning extracted from low temperature white light reflectance spectra on the planar, unprocessed wafer. The respective absorption dips can be related to the lower (LP), middle (MP) and upper (UP) polariton branches. The inset shows a section of the reflectance spectrum near resonance between the photon mode and the heavy hole QW-exciton. The Rabi-splitting between the LP and the MP branch determines to (8.7 ± 0.1) meV. (**b**) Power dependent emission intensity (red) and linewidth evolution (blue). (**c**) Power dependent emission energy of the relevant in-plane wave vector where condensation takes place around ~ $k_{||}$ = 1 µm^{-1}. Typical "S"-shape of the intensity accompanied by a sharp linewidth drop at a threshold power of (4.5 ± 0.5) mW in (b) in combination with the continuous blue shift in (c) are clear signatures for polariton condensation.

Exciton Density Estimate

We assume a coupling efficiency of the microscope objective of T_{Obj} = 0.8, a transmission of the cryostat window of T_{Cryo} = 0.9, and furthermore a sample reflectivity of R_{Sample} = 0.30 at the pumping wavelength extracted from low temperature reflectivity measurements. According to [2] one can assume 1% absorption per QW. With the given spot size of d_{Spot} = 5 µm and the excitation power of P = 20 mW at a laser energy of E_{Laser} = 1.664 eV one can calculate the exciton density per QW n_{Ex} via:

$$n_{Ex} = \frac{P \cdot T_{Obj} \cdot T_{Cryo} \cdot \left(1 - R_{Sample}\right) \cdot A_{QW}}{\pi \left(\frac{d_{Spot}}{2} \right)^2} \cdot \frac{\tau}{E_{Laser}}$$

According to [3] the relevant timescale is the average relaxation time of the excitons scattering into the polariton states. Therefore τ is in the range of 20 ps for the used GaAs QWs [4]. Thereby we get an exciton density of $3.9 \cdot 10^{10}$ cm^{-2} which is approximately one order of magnitude smaller than the expected Mott transition at $3 \cdot 10^{11}$ cm^{-2} in a GaAs QW system [5]. One can also estimate the polariton density via the observed blueshift of ~ 1 meV and the polariton-polariton interaction strength $\alpha \approx 6\, E_B\, a_B^2$ by using standard values for the exciton binding energy E_B and the exciton Bohr radius a_B [6]. Here we get a polariton density of ~ $2 \cdot 10^{10}$ cm^{-2}.

Time-resolved measurement

The spatial dependence of the polariton decay rate Γ(x) along the wire, especially close to the electric gate, can be accessed via time resolved spectroscopy. We excited the microwire at a various positions in the closed pinhole configuration non-resonantly in the first high-energy Bragg mode with a pulsed Ti:sapphire laser. The laser facilitates 50 ps long pulses with a repetition rate of 82 MHz. The corresponding PL signal has been filtered for the exciton line around 1.5957 eV, using the grating position of our spectrometer, and was measured with an avalanche photodiode (APD), connected to the side port of the spectrometer. The spectral bandwidth is given by the grating and the slit size of the side port and amounts to 150 µeV at the given energy. The APD provides a nominal time resolution of 40 ps. Since the exciton lifetime is associated with the polariton lifetime via the Hopfield coefficients and the quality factor, it is a good indication to measure the bias and spatial dependency of the exciton lifetime along the microwire sample to get an insight into Γ(x). The exciton lifetime has been obtained by measuring the actual exponential decay of the exciton reservoir that is built up in the duration of the 50 ps pulse.

Fig. S2a and b depict selected time-resolved measurements of the exciton decay at a distance of 7 µm away from the contact for different applied voltages (a) and at a constant bias of -1.8 V at different positions on the wire (b). In (a) one can see a clear evidence for a faster exciton decay which means a shorter lifetime with an increasing applied reverse bias. However, in (b) one can see a prominent spatial dependence of the exciton decay along the wire. Exponential fitting of the decay slope yields the exciton lifetime which is shown in Fig. S2c as a function of the position along the microwire for several applied voltages. The center of the 10 µm wide contact is located around 0 µm and its width is indicated by the vertical dotted lines. The increasing reverse bias results in a decreasing exciton lifetime and furthermore in a formation of a lifetime minimum right underneath the contact due to enhanced exciton ionization, introduced by the applied electric field. Just like the potential minimum underneath the contact, the lifetime minimum can be described by a Gaussian shaped function indicated by the solid lines. Note that the electric field leading to the exciton ionization typically splits up in a component perpendicular and parallel to the quantum well plane [7]. Both components lead to exciton field ionization but on different field strength scales [8]. Due to the pin-doping the in-plane component is suppressed and the component perpendicular to the quantum well plane should be the most relevant electric field component for the tunneling process.

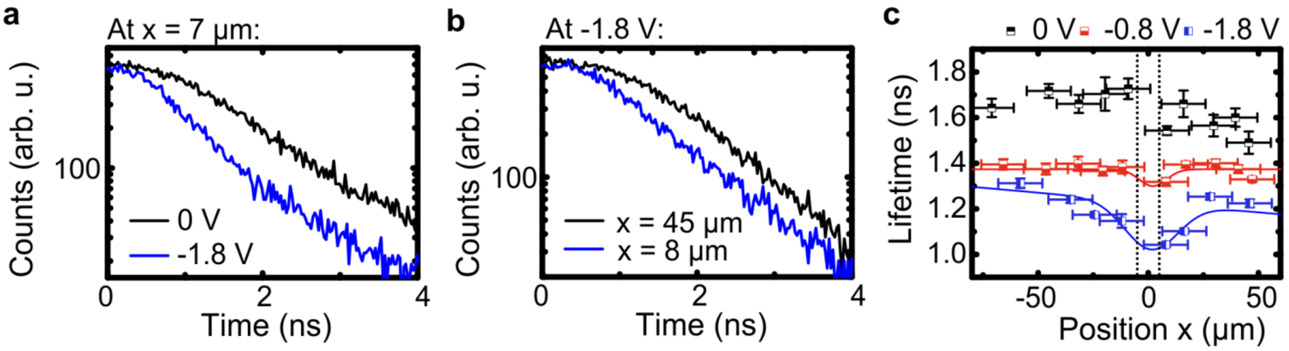

Figure S2 | Bias and spatial dependence of the exciton lifetime along the wire. (**a**) Time-resolved measurement of the exciton decay at a distance of 7 µm away from the contact for different applied voltages (0 V black; -1.8 V blue) in semi-logarithmic scale. With increasing reverse bias the excitons decay faster which leads to a shorter lifetime. (**b**) Time-resolved measurement of the exciton decay at a constant applied bias of -1.8 V at different positions along the wire in semi-logarithmic scale. The decay indicates a prominent spatial dependence of the exciton lifetime along the wire. (**c**) Exponential fitting of the respective decay slopes yields the spatially resolved exciton lifetime along the microwire sample for different applied voltages. An increasing reverse bias leads to a general reduction of the exciton lifetime and furthermore to the formation of a lifetime minimum underneath the contact. The minimum can be fitted by a Gaussian shape function (solid lines) and serves as a guide to the eye.

Cascadability

To satisfy cascadability, the output of one device must be able to control the output of a second one. For that purpose we extended the incoherently driven Gross-Pitaevskii approach to the case of two devices. While keeping the intrinsic potential gradient one can speak of the upper (1) and the lower switch (2) (cf. Fig. S3b). First of all, both devices can be independently switched between the two stable states of the bistable regime by the application of electric pulses. Remarkably, for electric pulses smaller than the width of the hysteresis region, the state of the lower switch (2) can only be switched if the upper switch (1) is in a specific state. Fig. S3a depicts the intensity at the contact corresponding to the lower switch (2) and upper switch (1). Initially both devices are in the high intensity state. At 2 ns a voltage pulse switches the upper switch into the lower intensity state. At 4 ns, a weak voltage pulse applied to the lower switch (2) is unable to switch its state. At around 8 ns the upper switch (1) is reset to the high intensity state. Due to its coupling to the lower switch this allows the lower switch to be switched when a weak pulse (same amplitude as the pulse at 4 ns) is applied at around 10 ns. This represents the electrical analogue of the cascadability shown by Ballarini et al. [9].

Logic-level restoration

The present system is ideal for logic-level restoration since there is a well-controlled bistability. The bistability is triggered by the carrier reservoir in the presence of the contact-induced trap. The bistable behavior vanishes as soon as the pump spot is moved away from the gate. Switching the device between the “off”- to the “on”-state is already sufficient to encode binary information. However, operating in the bistable regime means that the state of any device is necessarily in one of two stable distinguishable states which reduce the impact of noise on the system. Therefore, the quality of the logic signal should be restored at each stage when extending the experiment to the case of several devices and signal degradations should not propagate through the system.

Logic level independence of system losses

Working in the bistable regime allows encoding of binary information, where the two bistable states represent clearly distinguishable logic levels. Considering a system made with many devices, it is reasonable to assume that each switch would be defined by an identical hysteresis curve. Hence all switches throughout a circuit would be defined with the same logic-levels.

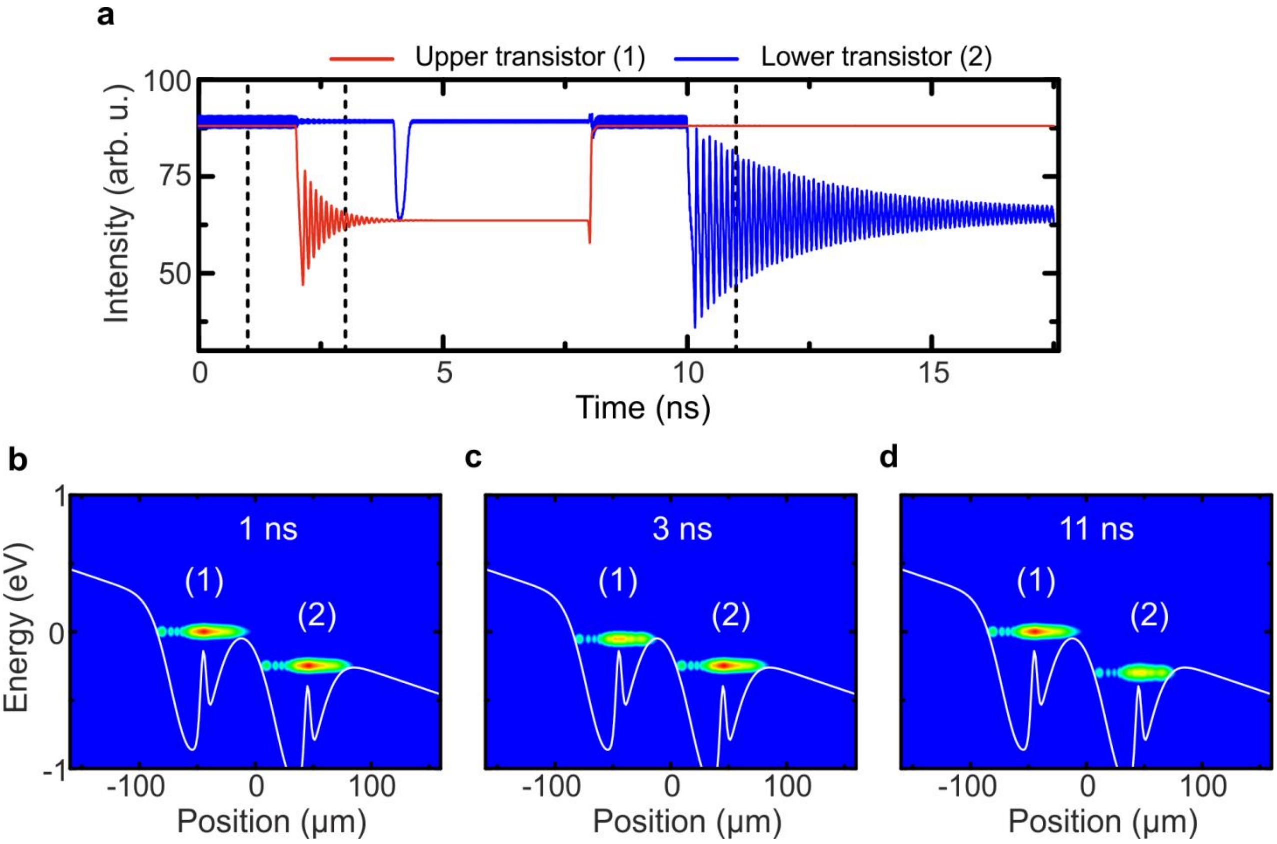

Figure S3 | Extended Gross-Pitaevskii model for cascadability. (**a**) Total integrated intensity at the contact of the corresponding upper (1) and lower switch (2). For weak voltage pulses, the state of the lower switch (2) can only be switched when the upper switch (1) is in a specific state. (**b-d**) Potential landscape for the injected polariton flow for the case of two devices at different times corresponding to the time evolution of (**a**). (**b**) At 1 ns both devices are switched to the on-state. (**c**) At 3 ns only the lower switch (2) is switched to the on-state while the upper switch is switched to the off-state. (**d**) At 11 ns the upper switch (1) is switched to the on-state and therefore offers the possibility to switch the lower switch (2) to the off-state.

The calculation shown in Fig. S3 demonstrate that it is possible for the state of one switch to affect logically the state of its neighbor, when the devices are operated in the bistable regime. Within this scheme there should be no loss of information as it propagates through a larger circuit. The reason is that although there are losses of polaritons as they propagate, they are fully compensated by the gain, coming from the incoherent driving of each switch region, when they arrive at the next device. Consequently, there is no need to define logic-levels based on information loss.

Amplification

Amplification of a polariton signals in one dimensional channels has already been demonstrated several times [10, 11] in configurations that can be implemented straight forwardly. While in the work of Wertz et al. and Niemietz et al. the amplification of the propagating polariton condensate has been accomplished by providing gain via an optically induced exciton reservoir, it is reasonable to assume that local electrical injection would accomplish the same task. Beyond that, for the case of two devices (see discussion of “cascadability”) we believe that amplification is already implemented since losses of polaritons are fully compensated by the gain, coming from the incoherent driving of an additional laser at each switch region.

References

1. Brodbeck, S. et al. Impact of lateral carrier confinement on electro-optical tuning properties of polariton condensates. *Appl. Phys. Lett.* **107,** 041108 (2015).

2. Masselink, W. T. et al. Absorption coefficients and exciton oscillator strengths in AlGaAs-GaAs superlattices. *Phys. Rev. B* **32,** 8027 (1985).

3. Bajoni, D. et al. Polariton light-emitting diode in a GaAs-based microcavity. *Phys. Rev. B* **77,** 113303 (2008).

4. Tempel, J.-S. et al. Characterization of two-threshold behavior of the emission from a GaAs microcavity. *Phys. Rev. B* **85,** 075318 (2012).

5. Deng, H., Weihs, G., Santori, C., Bloch, J. & Yamamoto, Y. Condensation of semiconductor microcavity exciton polaritons. *Science* **298,** 199-202 (2002).

6. Tassone, F. & Yamamoto, Y. Exciton-exciton scattering dynamics in a semiconductor microcavity and stimulated scattering into polaritons. *Phys. Rev. B* **59,** 10830 (1998).

7. Rapaport, R. et al. Electrostatic traps for dipolar excitons. *Phys. Rev. B* **72,** 075428 (2005).

8. Miller, D. A. B. et al. Electric field dependence of optical absorption near the band gap of quantum-well structures. *Phys. Rev. B* **32,** 1043 (1985).

9. Ballarini, D. et al. All-optical polariton transistor. *Nat. Commun*. **4,** 1778 (2014).

10. Wertz, E. et al. Propagation and amplification dynamics of 1D polariton condensates. *Phys. Rev. Lett.* **109**, 216404 (2012).

11. Niemietz, D. et al. Experimental realization of a polariton beam amplifier. *Phys. Rev. B* **93,** 235301 (2016).
